# Senescent cells, a promising target for Alzheimer's Disease

**DOI:** 10.1002/alz.086907

**Published:** 2025-01-03

**Authors:** Ana Guerrero

**Affiliations:** ^1^ Institute of Neurosciences, University of Barcelona, Barcelona, Catalunya Spain

## Abstract

**Background:**

Senescence is a cellular response to stress or damage leading to a state of irreversible growth arrest. As we age, the number of senescent cells increases and directly contributes to age‐related conditions including cancer and neurodegenerative diseases. As a result, there is a growing interest to therapeutically target senescence either with drugs eliminating senescent cells (senolytics) or with strategies to modulate their secretory phenotype among others. Importantly, even though the accumulation of senescent cells is a key hallmark of ageing, and ageing the biggest risk factor for Alzheimer´s Disease (AD), the extent of senescence in the brain during AD remains elusive.

**Method:**

We have developed a new preclinical model combining a mouse model of amyloid pathology (App NL‐G‐F knock‐in) with both a senescence transgenic reporter and a conditional targeted cell ablation lines based on *p16Ink4a* expression, a well‐known senescence marker. This model allows to track the onset of cellular senescence in response to amyloid deposits and to evaluate the therapeutic potential of the selective elimination of senescent cells.

**Result:**

In this Presentation, I will give an overview on the available strategies to therapeutically target senescence and share some preliminary data obtained with our new preclinical model.

**Conclusion:**

There is an urgent need to explore new therapeutic avenues to improve the lives of those affected by AD, either by delaying the onset and progression of the pathology, or by ameliorating the symptoms of this devastating disease. Cellular senescence constitutes a promising target that warrants further investigation.

